# Neuro-protective effect of rutin against Cisplatin-induced neurotoxic rat model

**DOI:** 10.1186/s12906-017-1976-9

**Published:** 2017-09-29

**Authors:** Mashal M. Almutairi, Wael A. Alanazi, Musaad A. Alshammari, Moureq Rashed Alotaibi, Ali R. Alhoshani, Salim Salah Al-Rejaie, Mohamed M. Hafez, Othman A. Al-Shabanah

**Affiliations:** Department of Pharmacology and Toxicology, College of Pharmacy, King Saud University, P.O. Box 2457, Riyadh, 11451 Kingdom of Saudi Arabia

**Keywords:** Gene expression, Real time PCR, Cisplatin, Oxidative stress, Rutin

## Abstract

**Background:**

Cisplatin is widely used chemotherapeutic agent for cancer treatment with limited uses due to its neurotoxic side effect. The aim of this study was to determine the potential preventive effects of rutin on the brain of cisplatin- neurotoxic rat model.

**Methods:**

Forty rats were divided into four groups. Group-1 (control group) was intra-peritoneal (IP) injected with 2.5 ml/kg saline. Group-2 (rutin group) was orally administrated 30 mg/kg rutin dissolved in water for 14 days. Group-3 (cisplatin group) was IP received 5 mg/kg cisplatin single dose. Group-4 (rutin and cisplatin group) was orally administrated 30 mg/kg rutin dissolved in water for 14 days with a single dose of 5 mg/kg cisplatin IP on day ten. Brain tissues from frontal cortex was used to extract RNA, the gene expression levels of paraoxonase-1 (PON-1), PON-2, PON-3, peroxisome proliferator-activated receptor delta (PPAR-δ), and glutathione peroxidase (GPx) was investigated by Real-time PCR.

**Results:**

Cisplatin significantly decreased the expression levels of *PON-1*, *PON-3*, *PPAR*-δ and *GPX* whereas significantly increased *PON-2* expression levels. Co-administration of Rutin prevented the cisplatin-induced toxicity by restoring the alteration in the studied genes to normal values as in the control group.

**Conclusion:**

This study showed that Rutin has neuroprotective effect and reduces cisplatin- neurotoxicity with possible mechanism via the antioxidant pathway.

## Background

Platinum-based compounds, such as cisplatin, are part of standard treatment for various cancers [1]. Cisplatin is an old drug approved by the Food and Drug Administration in 1978 [2, 3], then it becomes one of the most commonly prescribed anti-cancer drugs. Cisplatin causes cell-cycle arrest leading to apoptosis [4], but the core mechanism is not only its ability to covalently bind to DNA but also to a broad range of essential RNA molecules. Recent near atomic resolution study showed that cisplatin interacts with various RNA sites in the ribosome [5].

Cisplatin-related side effects (ototoxicity, nephrotoxicity, neurotoxicity and cerebral disorders) limits its clinical use at the desired dosage [6, 7]. Several studied have investigated the mechanisms of cisplatin toxicity but the mechanisms for induction of peripheral neuropathies is poorly understood [8–10]. One study showed the ability of cisplatin to penetrate into the brain where it inhibits neuronal stem cell proliferation [11]. Cisplatin-induced neurotoxicity leads to dose reduction or early termination of chemotherapy that can affect patient life [12, 13]. Cisplatin-induced neurotoxicity via oxidative damage, inflammation, mitochondrial dysfunction, DNA damage, and apoptosis in the nervous system [11, 12]. Cisplatin-induced neurotoxicity through the formation of nucleoli abnormalities in the spinal root ganglion cells of rat embryo [14, 15]. The cisplatin side effects on both human and animal nervous systems can be proven with electrophysiological and histopathological experiments [5, 7, 9]. Chronic cisplatin administration leads to severe damage in spinal ganglia neurons and decreases cell size [16] via interference of platinum with DNA synthesis [4].

Paraoxonase (PONs) is a multigene family composed of three members (PON1, PON2, PON3) coded for enzymes capable of hydrolyzing organophosphate compounds; and plays a role in inflammation and oxidative stress [17]. The enzymes of PONs have anti-atherogenic role through its ability to retard the oxidation of LDL [18]. PON1 is paraoxonase/arylesterase that hydrolyses a broad range of substrates and is a lactonase with lipophilic lactones substrates [19]. PON2 hydrolyses and inactivates N-acyl-homoserine lactones. *PON1* gene is expressed in brain [20] and *PON2* gene is expressed in lungs, heart, liver and brain, but is not detected in blood [21]. PON3 can hydrolyse bulky drug substrates, such as lovastatin and spironolactone [19]. PON1 and PON3 are synthesized in liver and are attached to high-density lipoproteins (HDL) in blood [22, 23].

Oxidative stress has an important role in toxicity produced by different drugs such as doxorubicin and cisplatin [24, 25]. Cisplatin produces oxidative stress through reduction of plasma antioxidant enzymes levels such as catalase, glutathione peroxidase and superoxide dismutase leading to a failure of the antioxidant defense against free radical damage generated by antitumor drugs [26]. DNA damage and inflammatory cytokines are major players in cisplatin-induced cytotoxicity [27]. The increased reactive oxygen species (ROS) react with DNA to permit the formation of 8-hydroxy guanine causing damage to DNA [28]. The excess generation of ROS increases the damage of biomolecules resulting in lipid peroxidation.

Antioxidants play a vital role in inhibiting the generation of free radicals subsequently preventing the oxidative damage. The antioxidants are naturally present in the body, while others have to be provided as supplements. Several antioxidant agent can reduce the cisplatin-induced cytotoxicity. Parsley oil, with its antioxidant activity, used in the treatment of cisplatin-induced hepatic and cardiac injuries [29]. Other study found that ceftriaxone displayed protective efficacy against cisplatin-induced renal tubule-interstitial fibrosis, possibly via anti-fibrotic potential [30]. Other natural product such as honey bee and royal jelly could be used as dietary preventive natural products against subchronic cisplatin-induced renal injury [31]. Flavonoids are poly-phenolic compounds with anti-inflammatory, antiviral, antibacterial, and neuroprotective properties [32].

Rutin, a flavonoid glycoside, found in vegetables, fruits, tea and herbs [33]. Moreover, rutin possess different protective effects including antioxidant, anti-cancer and anti-inflammatory properties [34]. Interestingly, several studies showed that rutin significantly reduced the cisplatin-induced oxidative stress via decreasing lipid peroxidation and increasing antioxidant activity [35–37]. Also, rutin has a protective effect against doxorubicin-induced memory deficits and has neuroprotective effects in streptozotocin-induced diabetes in rats [38, 39]. In addition, it has a protective function in ischemic organs including the heart and brain [40]. Rats are used as models of human disease because the rats provide many advantages over other organisms, including the size of their body and substructures in organs. In addition, the ability to measure drug effects at specific anatomical areas [41]. Therefore, this study aimed to investigate the possible protective effect of the rutin via studying some genes of the antioxidant pathway in the brain tissues of cisplatin-induced neurotoxic rat model.

## Methods

### Animals

The experiments were carried out on six-week-old male Wistar rats weighing 230– 260 g obtained from the Animal Care Center, College of Pharmacy, King Saud University, Riyadh, Saudi Arabia. The animals were kept under standard conditions of temperature (22 ± 1 °C), humidity (50–55%), and a 12-h light/dark cycle, with free access to standard laboratory feed and water, according to the study protocol. All methods were conducted according to the Guide for Care and Use of Laboratory Animals, Institute for Laboratory Animal Research, National Institute of Health (NIH publication No. 80–23; 1996). The Experimental Animal Care Center Review Board, college of pharmacy, King Saud University Riyadh, Saudi Arabia, approved the protocol included in this study (number E.A.C.B -5/2017).

### Chemicals

Cisplatin (1 mg/ml sterile concentrate) was a generous gift from King Khalid University Hospital drug store, King Saud University, Kingdom of Saudi Arabia. Rutin was purchased from Sigma Chemicals (Sigma-Aldrich Louis, MO, USA). Primers were designed using primer express 3 (Applied Biosystem, Life Technologies, Grand Island, NY, USA) and high capacity reverse transcriptase and Syber Green master mix kits were purchased from Applied Biosystems (Life Technologies, Grand Island, NY, USA).

### Methods

Experimental Design was followed Kamel et al., protocol [42]. In brief, 40 rats were randomly divided into four groups (ten rats each) and subjected to treatment as follows: Group-1 (control group) was IP injected with 2.5 ml/kg saline; Group-2 (rutin group) was orally (using Gavage) administrated 30 mg/kg rutin (dissolved in water) for 14 days; Group-3 (cisplatin group) was IP injected with 5 mg/kg cisplatin single dose [43, 44] and Group-4 orally administrated 30 mg/kg rutin dissolved in water for 14 days with a single dose of 5 mg/kg cisplatin IP on the day ten (rutin and cisplatin group).

At least 24 h after the last treatment protocol, all animals were weighted and were recorded after that the animals were anesthetic by exposed to ether according to our laboratory protocol and conducted in compliance with Institutional Animal Care and Use Committee policy, September 2013 (IACUC POLICY # 13) and killed by decapitation, during this procedure, the rats were unconscious [45]. The brain was immediately removed, washed with an ice-cold saline solution and then snap frozen in liquid nitrogen and finally stored until used for the molecular studies.

### Bioassays

#### Serum Thiobarbituric acid reactive substances (TBARS)

Lipid peroxidation, in brain tissues, was determined using TBARS assay kit (Cayman Chemical, MI) according to the manufacturer’s instructions. Briefly, MDA standard curve was prepared by diluting 250 μL MDA standard with 750 μL water and then serial dilution that started from 0 μm to 50 μm was prepared. A mixture of 100 μL of the serum sample, 100 μL of homogenate brain tissues in cold 10 mM Tris-HCl (pH 7.5), standard and 100 μL of SDS was first prepared. Four milliliters of color reagent was added to each mixture and boiled for an hour. After that, the reaction was stopped on ice for 10 min and centrifuged for 10 min at 1600×g; then 150 μL of the supernatant was loaded in a 96-well plate and absorbance was read at 540 nm. TBARS concentration was calculated from MDA standard curve.

##### Estimation of glutathione (GSH) levels in brain tissues

Glutathione concentration was determined by the previously described method by Sedlak and Lindsay [46]. Briefly, 0.2 mg brain tissues were homogenized in ice-cold 0.02 M EDTA then 0.5 ml of tissue homogenate was mixed with 0.2 M Tris buffer, pH 8.2 and 0.1 ml of 0.01 M Ellman’s reagent, [5,5′-dithiobis-(2-nitr-benzoic acid)] (DTNB). Each sample tube was centrifuged at 704×g at room temperature for 15 min. The supernatant was measured using spectrophotometer (LKB-Pharmacia, Mark II, Ireland) at 412 nm.

##### Determination of the genes expression levels in brain tissues:

Total RNAs were extracted from frontal cortex brain tissue by Trizol method according to the manufacturer’s protocol as previously described [47]. The RNA concentrations and purity were measured by NanoDrop (NanoDrop 8000, Thermo Scientific, USA). Total RNA was electrophorized on ethidium bromide-stained agarose gel. The isolated RNA has an A 260/280 ratio of 1.9–2.1.

#### cDNA synthesis and real-time PCR methods

First-strand cDNA was synthesized from 1 μg of total RNA by reverse transcription using high capacity reverse transcriptase kit (life technology, Applied Biosystem, USA) according to the manufacturer’s instructions. Real-time PCR was done using 2^-ΔΔC*t*^ method according to our previous study [48]. GAPDH gene was used as endogenous control. All primers used in this study were synthesized in Jena Bioscience Germany and were listed in Table 1. Following amplification, melting curve analysis was performed to verify the correct product according to its specific melting temperature (Tm).

**Table 1:** The primers sequences that used in this study

Gene Name	Forward primer	Reverse primer
PON-1	5′-TGAGAGCTTCTATGCCACAAATG-3′	5′-CCATGACAGGCCCAAGTACA-3′
PON-3	5′-CATCCAGGATCCTTTGTCAGATAA3’	5′-CACGGTGCTGCCCTGAAG-3’
PON-2	5′-ACGGCCAGAAGCTCTTCGT-3’	5′-TCTCGGATAGAATGTTCTGAATTCG-3’
PPAR-δ	5′-GCCAAGAACATCCCCAACTTC3’	5′-GCAAAGATGGCCTCATGCA-3’
GPx	5′-CGGTTTCCCGTGCAATCAGT3’	5′- ACACCGGGGACCAAATGATG-3’
GAPDH	5′-AACTCCCATTCCTCCACCTT-3’	5′-GAGGGCCTCTCTCTTGCTCT-3’

#### Statistical analysis

Differences between obtained values (mean ± SEM, *n* = 10) were carried out by one-way analysis of variance (ANOVA) followed by the Tukey-Kramer multiple comparison test. A *P* value of 0.05 or less was taken as a criterion for a statistically significant difference using graph pad 5.0 prism software (GraphPad Software, Inc., La Jolla, CA, USA).

## Results

### Rutin restores rat body weight, TBAR, and GSH levels

**Fig. 1:**
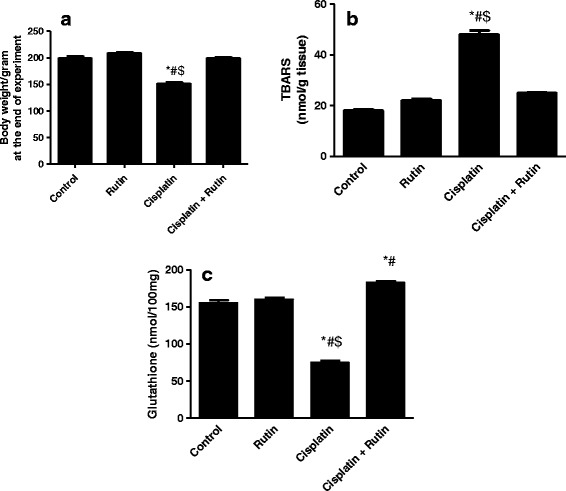
The Effect of Cisplatin, rutin, and their combination on the body weight (**a**), TBARs levels (**b**) and glutathione (**c**). Data were presented as mean ± SEM (*n* = 10). *, # and $ indicate significant change from control, rutin and CP + rutin, respectively, at *P* < 0.05 using ANOVA followed by Tukey–Kramer as a post ANOVA test

The effect of cisplatin, rutin and their combination on the rat body weight during the experiment was shown in Fig. 1a. The injection of cisplatin did not kill any rat during the whole experiment. However, following the injection, the animals significantly lost body weight compared to a steady weight gain of the controls.

Cisplatin induces oxidative stress, which in turn cause lipid peroxidation. This effect can be studied by determining the level of the lipid degradation product such as TBAR. Therefore, we studied if rutin can protect animal brain tissues against lipid peroxidation. As expected cisplatin increased TBAR levels significantly in brain tissues compared to control group by 375% (*P* < 0.001) indicating an increase in the free radicals (Fig. 1b). However, combining cisplatin with rutin reversed the increase in TBAR level to normal values as in the control group (Fig. 1b). Thus, these results showed that rutin could prevent the cisplatin-induced lipid peroxidation and neuroprotective the cell.

Glutathione (GSH) is natural antioxidant that presents almost in all domains of life and its availability in normal level protects cells from ROS. Our study showed clearly that cisplatin decreased the GSH levels by 50% in comparison with the control group. Interestingly, rutin administration in combination with cisplatin was able to increase GSH level to its normal level (Fig. 1c). These results showed that cellular oxidative damage caused by cisplatin through affecting the level of GSH could be prevented by co-administration of rutin.

### Rutin increases *GPx* expression to normal range in brain tissues

**Fig. 2:**
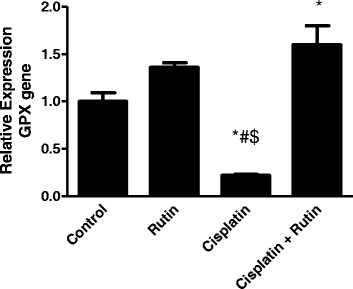
The Effect of Cisplatin, rutin, and their combination on the expression levels of Glutathione peroxidase in rat brain. Data were presented as mean ± SEM (*n* = 10). *, # and $ indicate significant change from control, rutin and CP + rutin, respectively, at *P* < 0.05 using ANOVA followed by Tukey–Kramer as a post ANOVA test

Glutathione peroxidase (GPx) is one of the most crucial antioxidant enzymes. In this study we interested in examining the effect of cisplatin on GPx expression level in brain tissues of rats model. Cisplatin treatment reduced the GPX expression level by 4.5-fold compared to control group (*p* < 0.05) (Fig. 2). The supplementation of rutin with cisplatin overexpressed GPX level by 6.3-fold compared to the cisplatin group (*p* < 0.02) (Fig. 2). These data illustrate the importance of rutin as protective agent of cisplatin-induced oxidative damage.

### Rutin promotes the normal expression of antioxidant genes in brain tissues

**Fig. 3:**
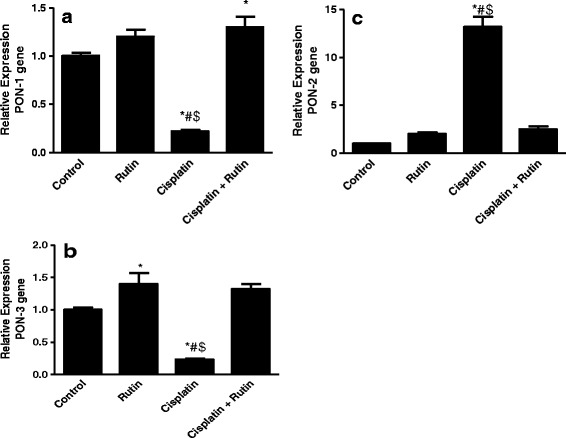
The Effect of Cisplatin, rutin, and their combination on the expression levels of PON-1 (**a**), PON-2 (**b**) and PON-3 (**c**) in rat brain tissues. Data were presented as mean ± SEM (*n* = 10). *, # and $ indicate significant change from control, rutin and CP + rutin, respectively, at *P* < 0.05 using ANOVA followed by Tukey–Kramer as a post ANOVA test

One of the enzyme families that has a role in the prevention of oxidative stress is PONs. In order to examine the effect of cisplatin on these genes, we analyzed their expression profile in vivo after exposure to cisplatin. Administration of cisplatin significantly decreases the *PON-1* and *PON-3* expression level by 4-fold (*p* < 0.05) and by 4.5-fold (*p* < 0.05), respectively, compared to control group (Fig. 3a and c). Interestingly, administration of rutin in combination with cisplatin completely restored *PON-1* and *PON-3* expression to their normal levels as in the control group (Fig. 3a and c). These reversal changes result in significant increase in the *PON-1* and *PON-3* expression level by 5.2-fold (*p* < 0.01) and 6-fold (*p* < 0.002), respectively, compared to cisplatin group and by 1.3-fold and 1.4-fold compared to control group (*p* < 0.5), respectively (Fig. 3a and c). Taken together, these results suggest that rutin neuroprotects the cells from cisplatin-induced stress by promoting the expression of *PON-1* and *PON-3* antioxidant genes*.*

As respond to increase in ROS, *PON-2* expression increases to antagonize oxidative stress. Therefore, in this study, we examine the effect of cisplatin on *PON-2* expression level. As shown in (Fig. 3b) the administration of cisplatin resulted in significant increase in the expression level of *PON-2* by 12-fold (*p* < 0.001) compared to control. Strikingly, combining rutin with cisplatin resulted in a complete reversal of *PON-2* to their normal values as in the control group. These reversal changes result in significant decrease in the *PON-2* expression level by 5-fold (*p* < 0.5) compared to cisplatin group (Fig. 3b). These results demonstrate that rutin by itself can counteract the production of ROS by cisplatin, therefore, no need to elevate *PON-2*.

### Rutin reverses the effect of Cisplatin on PPAR-δ expression in brain tissues

**Fig. 4:**
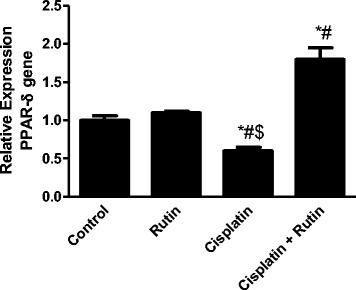
The Effect of Cisplatin, rutin, and their combination on the expression levels of PPAR-δ in rat brain. Data were presented as mean ± SEM (*n* = 10). *, # and $ indicate significant change from control, rutin and CP+ rutin, respectively, at *P* < 0.05 using ANOVA followed by Tukey–Kramer as a post ANOVA test

Activation of PPAR-δ expression reduces the intracellular ROS accumulation. We investigate the effect of cisplatin on the antioxidant mechanism and hence induce oxidative stress. Exposing the rats to cisplatin resulted in significant decrease in PPAR-δ expression level by 1.66-fold (*p* < 0.05) compared to the control group (Fig. 4). However, complementing cisplatin with rutin increases PPAR-δ expression level by 1 .8-fold (*p* < 0.02) and 3-fold (*p* < 0.01) compared to control and CP groups, respectively. These data showed that rutin could restore the antioxidant PPAR-δ expression resulting in protection of the cells from cisplatin-induced oxidative damage.

## Discussion

Cisplatin is a wildly used anticancer drug but its toxicities have limited its uses in cancer treatment at effective doses. Cisplatin causes lipid membrane peroxidation by increasing free oxygen radicals and reducing antioxidant production, finally resulting in extensive tissue damage [49]. Several mechanisms are proposed for the cisplatin-neurotoxicity in which oxidative damage is one of the important mechanism in cisplatin and other chemotherapeutic agents’ toxicity. The oxidative stress alters the cell structure and function, and reduces the antioxidant mechanisms resulting in DNA damage in biological systems [50]. Combining the drug with another protective agent is one of the methods used to decrease the severity of the drug-related toxicity. Several studies conclude that the antioxidant agent such as rutin, L-acetayle carnitine, Parsley oil, ceftriaxone, honey bee and royal jelly have antioxidant activity against chemotherapy [29–31, 51, 52]. The current study investigated the protective effect of rutin on the brain of the rat against cisplatin-induced neurotoxicity via studying the gene expression level of some genes related to the antioxidant pathway.

The neurotoxic effect was determined by measuring the TBAR, GSH and antioxidant genes levels in the brain tissue of the rat. In this study, administration of cisplatin significantly increase the TBAR and decline the GSH levels. Similarly, Turan and coworker found that cisplatin-induced oxidative stress in the brain tissue via significantly increased the TBAR and reduced the GSH levels [53]. The elevated TBARS levels in tissues indicate the increase in the free oxygen radical that results in cells destruction [54]. Glutathione provides the first line of defense against oxidative damage and toxic compounds and has role in several metabolic processes [55]. The decrease in the glutathione levels leads to reduction in the NADPH or GSH utilization in exclusion of peroxides [56].

Previous studies demonstrated that antioxidant agents could prevent cisplatin-induced neurotoxicity [43, 44]. Rutin is a potent bioflavonoid with powerful antioxidant, anti-cancer and anti-inflammatory properties [34]. In the current study, rutin co-administration with cisplatin reversed the changes in TBAR and GSH to their normal levels as in control group. Therefore, rutin may prevent lipid peroxidation on the cell membrane by scavenging the free oxygen radicals.

The oxidative stress can cause cell damage when losing the imbalance between ROS production and antioxidant defense [57]. In the brain, PONs are important in nerves myelination due to their protective function against lipid oxidation. PON-1 and PON3 are expressed in liver, and their protein products are associated with high-density lipoproteins in plasma. PON-1 and PON3 can protect LDL from oxidation by scavenging free radicals [46]. The antioxidant activity of PON1 is via its association with its –SH group that can affect its activity [58]. The inhibition of PON-1 expression and activity plays a role in neurotoxicity and oxidative stress [59]. In the brain, PON1 polymorphisms rs662 and rs854560 is involved in Alzheimer’s disease neuropathology [60]. The decrease in PON1 and PON3 expression levels is associated with toxicity induced by oxidative stress. Similarly, in our previous study, the decrease in PON1 and PON3 expression levels is associated with hepatotoxicity induced by carbon tetrachloride [61]. In the present study, rutin co-administration with cisplatin reverses the alteration in *PON1* and *PON3* expression levels and increases its antioxidant activity. Rutin reduces neurotoxicity via antioxidant activity. Previous study found that the neuroprotective effect of rutin in the rat brain ischemia was through its ability to reduce TBARS, H_2_O_2_ and GSH in the hippocampus and frontal cortex in the middle cerebral artery occlusion. In addition to its ability to reduce the expression of p53 and increasing of antioxidant enzymatic activities [40].

PON2 is a member of paraoxonase family [62] and is a ubiquitously expressed intracellular enzyme [63, 64]. PON2 mRNA and protein are detected in the brain [64, 65]. PON2 exerts an antioxidant effect and play a major role in neuroprotection [66, 67]. PON2 is localized primarily in the mitochondria [20, 68] and this support its role in protecting cells from oxidative damage. In the current study, cisplatin significantly increased the PON2 expression levels. Rutin administration decreases the expression levels of PON2 as in control group. Similarly, PON2 high expression is accompanying with resistance to oxidative stress-induced toxicity and may be one of its neuroprotective mechanisms [69]. The previous study showed that rutin has a neuroprotective effect in the brain ischemia in rats [40]. It also ameliorated morphological damage and attenuated ischemic neural apoptosis by reducing the p53 expression and increasing of antioxidant enzymatic activities [40].

The PON2 over-expression by cisplatin might be associated with increased the cells’ ability to scavenge ROS and to antagonize oxidant-induced toxicity. Other study found that the macrophage PON2 expression and activity were increased under oxidative stress and suggested that this increase might be a compensatory mechanism against oxidative stress [18].

Among the PPAR isoforms, PPARδ expression is abundant in the neural cell types and might play a role in the brain physiological functions [70] but its exact roles needs more clarification. The activation of PPARδ induced by a neurotransmitter involved in neurological disorders such as Alzheimer’s disease [71] and reduced the intracellular ROS accumulation. In the present study, cisplatin-induced reduction in the PPARδ expression level and this alteration was reversed by administering rutin. Similarly, previous study showed that PPARδ activation could induce antioxidant systems [72] as well as provide neuroprotection [73, 74].

## Conclusion

In conclusion, rutin showed neuroprotective effect on the brain of rat cisplatin- neurotoxic model with a possible mechanism via the antioxidant pathway.

## Abbreviations

ANOVA: One-way analysis of variance
cDNA: Complementary deoxynucleic acid
CP: Cisplatin
DNA: Deoxynucleic acid
GPX: Glutathione peroxidase (GPx)
GSH: Glutathione
HDL: High density lipoproteins
IP: Intra-peritoneal
LDL: Low density lipoproteins
NADPH: Nicotinamide Adenine Dinucleotide Phosphate-oxidase
PON-1: Paraoxonase-1
PON-2: Paraoxonase-2
PON-3: Paraoxonase-3
PPAR-δ: Peroxisome proliferator-activated receptor delta
RNA: Ribonucleic acid
ROS: Reactive oxygen species
TBARS: Thiobarbituric acid reactive substance
